# Supplementary material for: Metoclopramide and Propofol to Prevent Nausea and Vomiting during Cesarean Section under Spinal Anesthesia: A Randomized, Placebo-Controlled, Double-Blind Trial
Source: J Clin Med. 2021 Dec 26;11(1):110. doi: 10.3390/jcm11010110 (PMC8745529; doi:10.3390/jcm11010110)
Supplement: Supplementary file 1 [file jcm-11-00110-s001.zip › jcm-1498397-supplementary.pdf]

Supplement 1

Maternal Satisfaction Questionnaire

SCALA DI SODDISFAZIONE MATERNA

Questionario con punteggio da 1 a 7 (1= decisamente no, 7=decisamente si) e VAS della soddisfazione rispetto all'anestesia e all'andamento dell'intervento (da 0 a 10).

Valuti l'intensità del dolore durante l'intervento cesareo.		*Prurito?*	
Pensa che l'anestesia eseguita sia stata sicura per lei?		***Durante l'intervento in sala operatoria era capace di***	
Pensa che l'anestesia eseguita sia stata sicura per il suo bambino.		*Interagire?*	
Ha avuto dolore quando l'ago è stato inserito nella sua schiena.		*Interagire col bambino?*	
L'ago è stato inserito facilmente nella sua schiena?		*Avere un senso di controllo di sè?*	
Si trovava in una posizione confortevole quando l'ago è stato inserito nella sua schiena.		*Comunicare con lo staff?*	
Durante l'intervento ha provato		*Vedere il bambino dopo il parto?*	
Brivido?		*Tenere il bambino dopo il parto?*	
Secchezza della labbra o della bocca?		*Sapeva cosa lo staff stava facendo durante l'intervento?*	
Secchezza della gola?		*C'era un'atmosfera confortevole?*	
Un cambiamento del comportamento?		*E' stata capace di allattare il suo bambino dopo il parto?*	
Dopo l'intervento ha avuto		*Si è ripresa velocemente dopo l'intervento?*	
Problemi alla schiena?		***VAS complessivo soddisfazione***	

16. Morgan, P.J.; Halpern; S.; McCulloch; J.L. Comparison of maternal satisfaction between epidural and spinal anesthesia for elective cesarean section. Can J Anaesth 2000, 47(10), 956-61. doi: 10.1007/BF03024865.

GROUP	Valuti l'intenStà del dolore durante l'intervento cesareo.	Pensa che l'anesteSa eseguita Sa stata Scura per lei?	Pensa che l'anesteSa eseguita Sa stata Scura per il suo bambino.	Ha avuto dolore quando l'ago è stato inserito nella sua schiena.	L'ago è stato inserito facilmente nella sua schiena?	S trovava in una poSzione confortevole quando l'ago è stato	Brivido?	Secchezza della labbra o della bocca?	Secchezza della gola?	Un cambiamento del comportamento?	Problemi alla schiena?	Prurito?	Interagire?	Interagire col bambiN?	Avere un senso di controllo di sè?	Comunicare con lo staff?	Vedere il bambiN dopo il parto?	Tenere il bambiN dopo il parto?	Sapeva cosa lo staff stava facendo durante l'intervento?	C'era un'atmosfera confortevole?	E' stata capace di allattare il suo bambiN dopo il parto?	S è ripresa velocemente dopo l'intervento?	VAS complesSvo soddisfazione	MSQ
M	1	7	7	3	7	5	1	7	7	1	5	1	7	7	7	7	7	5	7	7	6	6	10	118
M	1	7	7	2	7	7	7	7	7	1	1	1	7	7	7	7	7	7	7	7	7	7	10	125
M	4	5	7	6	6	6	1	6	6	5	4	1	6	6	5	5	6	3	6	6	5	5	8	110
M	7	7	7	7	2	7	4	7	2	1	1	1	7	7	7	7	7	2	7	7	6	4	8	114
M	7	7	7	1	7	7	7	7	7	1	1	1	7	7	7	7	7	7	7	7	7	5	10	128
M	1	7	7	7	7	3	1	7	7	7	1	1	5	1	7	5	7	7	4	5	7	7	9	111
M	1	7	7	1	7	7	1	7	7	1	1	1	7	7	7	7	7	7	5	4	7	7	8	113
M	1	6	7	1	7	5	7	4	2	2	1	1	3	4	3	5	5	4	6	7	7	6	5	94
M	1	7	6	1	7	7	5	6	6	2	1	1	7	7	7	7	7	7	7	7	2	5	9	113
M	5	7	4	6	6	6	7	7	7	7	1	1	6	6	7	4	6	4	4	7	1	5	5	114
M	1	7	5	1	7	7	6	7	7	6	1	1	7	6	6	7	7	1	5	7	1	7	6	110
M	1	7	7	4	4	6	7	7	6	1	1	5	7	7	7	7	7	7	7	7	7	7	10	126
M	7	6	5	4	6	5	5	7	7	1	1	1	7	7	7	7	7	1	5	7	7	5	7	115
M	1	6	6	5	6	7	1	6	6	7	1	1	7	7	7	4	7	7	5	7	7	7	9	118
M	3	1	1	7	7	7	1	7	7	1	7	1	7	7	4	7	7	5	7	7	4	7	9	112
M	1	7	7	2	7	7	1	1	1	1	1	7	7	7	7	7	7	2	5	7	1	3	5	96
M	3	7	7	1	7	7	1	5	5	1	1	1	7	7	7	7	7	7	5	7	7	5	8	112

M	2	7	7	2	7	1	6	7	7	5	1	1	7	7	5	7	6	4	6	7	7	7	9	116
M	1	7	7	3	7	2	7	7	2	7	1	1	5	5	4	5	7	1	6	5	2	4	8	96
M	2	7	7	3	7	5	4	3	2	1	1	1	7	7	7	7	7	1	7	7	6	6	9	105
M	1	7	7	5	1	3	7	6	6	2	1	1	5	7	5	5	7	7	5	7	5	5	8	105
M	1	7	7	1	7	1	3	6	6	7	1	7	5	4	5	6	3	3	6	7	5	6	8	104
M	4	7	7	2	7	7	7	5	5	1	1	1	7	7	7	7	4	4	7	7	7	5	8	116
M	6	6	6	1	6	6	6	5	5	6	1	1	7	7	7	7	6	2	6	7	7	1	7	112
M	1	7	7	1	7	7	1	5	4	5	1	1	6	7	6	6	6	1	5	7	1	5	7	97
M	4	6	6	2	5	5	1	3	3	1	1	1	6	7	6	7	7	7	5	6	3	5	7	97
M	1	5	5	2	7	7	4	1	1	6	3	1	7	7	5	6	7	5	7	7	7	5	9	106
M	1	6	6	3	7	7	5	6	6	2	1	1	5	7	6	5	7	3	4	7	5	7	9	107
P	2	7	7	2	7	6	3	6	1	1	4	1	7	6	6	7	6	1	7	7	7	5	10	106
P	2	7	7	6	7	2	4	7	7	2	2	1	7	7	7	7	7	5	7	7	7	5	10	120
P	7	7	7	1	6	7	1	7	7	2	1	1	7	7	7	7	7	7	7	5	7	7	9	122
P	1	7	7	3	6	6	5	7	7	1	2	1	6	6	6	7	7	7	6	7	7	7	10	119
P	1	7	7	2	7	7	7	6	7	1	1	1	7	7	7	7	7	7	5	7	7	4	7	119
P	3	7	7	1	2	6	3	4	1	1	1	1	7	7	7	7	7	7	7	7	6	7	10	106
P	2	6	6	1	7	4	1	3	3	1	1	1	7	7	7	7	7	7	2	7	7	5	7	99
P	1	7	7	1	7	7	1	7	7	1	2	1	7	6	7	7	7	7	4	7	6	7	9	114
P	6	7	7	3	6	6	1	1	1	1	1	1	7	7	7	7	7	7	1	7	3	4	8	98
P	1	7	7	7	7	7	2	1	1	1	1	1	7	7	6	7	7	7	7	7	7	7	10	112
P	1	7	7	2	7	3	1	1	1	1	7	3	5	7	6	7	7	7	7	7	7	5	9	106
P	4	7	7	4	6	6	1	7	1	1	1	1	7	6	6	7	7	5	6	6	6	6	9	108
P	5	7	7	7	4	1	2	1	1	1	1	5	7	7	6	7	7	7	1	6	1	2	2	93
P	4	7	7	2	7	3	1	1	1	1	1	5	7	7	5	7	7	7	7	7	7	7	9	108
P	1	7	7	4	7	7	2	4	4	1	1	2	7	7	7	7	7	3	6	7	6	4	10	108
P	1	7	7	2	7	2	1	1	2	1	1	1	7	7	7	7	7	7	1	6	7	7	9	96
P	1	7	7	2	7	7	2	1	1	1	1	6	7	7	7	7	7	6	5	7	7	7	10	110

P	2	7	7	2	7	7	2	7	5	1	1	1	7	7	7	7	7	5	7	7	2	7	8	112
P	1	7	7	2	7	7	6	7	7	1	1	1	7	7	7	7	7	7	7	7	7	7	10	124
P	3	7	7	2	7	7	6	6	6	2	1	1	7	7	5	7	7	7	7	7	7	7	9	123
P	2	6	6	3	7	6	5	7	7	5	4	1	7	7	6	7	7	7	6	7	7	6	10	126
P	3	7	7	2	7	5	7	2	2	1	3	1	7	7	7	7	1	1	6	7	7	7	9	104
P	2	6	6	5	7	6	1	2	2	1	1	5	6	6	6	7	7	5	5	6	2	4	8	98
P	2	6	6	4	6	6	1	4	4	7	1	1	6	6	6	6	7	7	5	6	7	5	9	109
P	2	7	7	1	7	4	7	7	7	1	6	1	7	7	6	7	7	1	4	7	7	6	8	116
P	1	7	7	1	7	7	1	1	1	1	1	1	7	7	7	7	7	7	1	7	7	7	10	100
P	2	7	7	2	7	6	2	5	1	1	3	2	6	7	6	7	7	7	6	7	6	5	9	109
PLACEBO	2	7	7	7	7	7	7	7	1	1	1	1	7	7	7	7	7	7	7	7	1	7	8	119
PLACEBO	3	6	6	2	6	7	1	7	7	7	2	2	7	7	6	7	7	7	7	7	1	6	9	118
PLACEBO	1	7	7	2	7	7	7	7	7	7	7	1	7	7	7	7	7	6	2	7	1	1	8	119
PLACEBO	1	7	7	2	2	6	7	1	1	1	1	6	7	7	7	7	7	1	7	7	7	7	9	106
PLACEBO	1	7	7	2	7	5	6	4	4	4	2	1	7	7	7	7	6	6	6	6	5	4	8	111
PLACEBO	1	6	6	4	6	3	6	7	7	4	6	1	7	7	7	7	2	2	4	6	2	3	7	104
PLACEBO	4	7	7	1	7	4	4	1	1	1	1	1	7	7	7	7	7	7	7	7	7	7	9	109
PLACEBO	1	7	7	1	7	7	7	7	7	1	1	1	7	7	7	7	7	2	7	7	6	6	8	117
PLACEBO	1	7	7	3	6	7	2	6	5	1	1	1	7	7	7	7	7	5	7	7	7	7	9	115
PLACEBO	5	7	7	2	7	7	1	7	7	1	5	1	7	7	7	7	7	7	7	7	7	7	8	127
PLACEBO	2	7	7	1	7	6	4	5	5	2	4	1	7	7	7	7	7	7	4	7	7	7	8	118
PLACEBO	1	7	7	1	7	7	2	7	6	1	1	1	7	7	7	7	7	7	7	7	6	5	9	115
PLACEBO	1	5	5	7	4	4	1	2	2	1	1	1	6	7	6	6	6	6	7	6	7	7	8	98
PLACEBO	1	6	6	1	7	7	7	4	4	1	1	1	7	7	7	7	7	7	7	7	7	7	8	116
PLACEBO	1	7	7	7	4	7	1	1	2	7	1	1	7	7	7	7	7	1	1	7	1	6	10	97
PLAC	5	4	4	2	6	6	6	1	1	1	6	1	6	6	6	6	7	7	5	6	7	6	8	10

EBO																							5
PLACEBO	2	7	7	4	6	6	6	1	1	1	4	1	7	7	7	7	7	7	6	6	7	7	9	114
PLACEBO	1	7	7	2	2	6	7	1	1	1	1	1	6	7	6	6	7	7	7	7	6	6	8	102
PLACEBO	1	7	7	2	7	7	3	6	6	1	1	1	6	7	6	6	7	4	6	6	6	5	8	108
PLACEBO	1	7	7	1	7	7	7	6	6	1	7	1	6	7	6	6	7	7	5	7	4	2	7	115
PLACEBO	3	6	6	2	5	6	1	6	6	7	2	2	6	7	6	6	7	7	7	7	4	6	8	115
PLACEBO	1	7	7	5	4	7	1	1	2	7	1	1	7	7	7	7	7	2	3	7	2	6	10	99
PLACEBO	7	4	4	1	7	7	4	2	2	1	1	1	7	7	7	7	7	7	7	7	7	7	9	111
PLACEBO	3	7	7	1	7	7	7	7	7	2	1	1	7	7	7	7	7	7	7	7	7	7	8	127
PLACEBO	5	7	7	2	5	6	1	6	6	1	5	1	6	1	6	6	7	7	6	7	6	7	8	111
PLACEBO	5	6	6	3	6	6	6	2	2	2	2	2	7	7	6	7	7	1	6	6	2	6	6	103
PLACEBO	5	7	7	2	1	1	1	7	7	1	1	1	7	7	7	7	7	7	7	7	7	5	9	109
PROMET	1	7	7	2	7	7	1	1	1	7	1	1	7	7	5	7	7	7	1	7	1	1	10	93
PROMET	2	7	5	2	7	6	1	5	1	6	1	1	7	6	4	7	7	7	5	5	?	6	8	98
PROMET	1	7	7	7	7	7	7	7	7	7	1	1	6	7	7	6	7	7	7	7	4	6	10	130
PROMET	4	7	7	1	6	5	1	7	1	3	7	1	6	7	5	7	6	7	5	7	6	4	7	110
PROMET	3	7	7	1	7	7	1	1	1	1	1	1	7	7	7	7	7	7	7	7	7	7	10	108
PROMET	6	7	6	3	3	3	7	5	4	7	7	1	5	5	2	4	7	4	4	6	7	5	7	108
PROMET	1	7	7	6	5	7	7	1	1	1	1	1	7	1	7	7	7	7	7	7	7	7	7	109
PROMET	1	7	7	2	7	1	3	1	1	2	6	1	7	7	7	7	7	6	6	7	5	7	9	105
PROMET	2	7	7	3	7	5	1	1	1	1	1	1	7	7	6	7	7	7	7	7	7	7	10	106
PROMET	1	7	7	3	6	2	1	7	1	7	1	1	7	7	7	7	7	7	7	7	7	5	9	112
PROMET	1	3	3	1	7	7	7	4	4	1	1	1	7	7	7	7	7	7	7	7	4	4	7	104
PROMET	1	7	7	5	7	7	3	1	1	3	2	1	7	7	7	7	7	7	7	7	2	1	10	104
PROMET	1	7	7	1	7	7	7	7	4	7	1	1	7	7	7	7	7	7	7	7	7	5	8	125
PROM	1	7	7	1	6	7	5	6	6	2	1	1	7	7	4	7	7	5	7	7	2	5	9	10

ET																								8
PROMET	1	4	4	1	1	1	1	7	4	1	1	1	7	7	4	7	7	1	7	4	7	6	8	84
PROMET	2	7	7	4	6	6	7	7	7	1	1	1	7	7	7	7	7	7	6	7	1	1	8	113
PROMET	1	6	6	6	1	6	1	1	1	1	1	1	7	7	7	7	7	5	7	7	7	7	9	100
PROMET	1	7	7	1	7	7	1	6	1	1	1	1	7	7	7	7	7	7	4	7	7	5	9	106
PROMET	1	3	3	2	5	2	4	1	1	1	1	1	7	7	7	7	7	7	6	6	7	7	10	93
PROMET	2	7	7	1	6	4	1	1	2	1	6	1	7	7	6	7	7	7	5	7	7	6	9	105
PROMET	1	7	7	2	6	2	2	2	2	1	5	1	7	7	6	6	7	7	5	7	5	7	9	102
PROMET	1	7	7	5	5	6	1	1	1	1	3	1	7	7	7	7	7	7	5	7	5	7	10	105
PROMET	4	7	7	1	6	5	1	1	1	1	2	1	7	7	7	7	7	6	6	7	5	7	10	103
PROMET	5	7	7	2	5	5	1	7	7	2	5	1	6	7	6	6	7	7	4	7	5	5	8	114
PROMET	1	7	7	2	6	7	1	3	3	7	1	1	7	7	5	7	7	7	5	7	4	6	9	108
PROMET	1	1	6	6	1	6	6	1	1	7	2	1	1	7	7	7	7	7	6	7	2	6	9	96
PROMET	1	7	7	2	7	7	1	6	6	1	1	1	7	7	7	7	7	7	7	7	7	7	10	117
PROMET	1	7	7	1	6	6	6	7	7	1	1	1	7	7	7	7	7	2	6	7	1	7	9	109

Supplement 2 : Blood pressure data, systolic > 90, diastolic > 50.

GROUP	SAP PRE	DAP PRE	SAP post S.A	DAP POST S.A.	SAP AFTER UMB	DAP AFTER UMB	SAP UTERINE	DAP UTERINE	SAP END	DAP END	SAP PRE	SAP post S.A	SAP AFTER UMB	SAP UTERINE	SAP END	DAP PRE	DAP POST S.A.	DAP AFTER UMB	DAP UTERINE	DAP END
M	108	70	112	69	115	63	100	59	105	65	0	0	0	0	0	0	0	0	0	0
M	114	74	73	36	106	68	114	58	96	51	0	1	0	0	0	0	1	0	0	0
M	130	87	130	87	118	74	88	68	117	80	0	0	0	1	0	0	0	0	0	0
M	149	92	134	64	139	75	136	75	144	78	0	0	0	0	0	0	0	0	0	0
M	115	84	135	77	144	89	103	60	106	81	0	0	0	0	0	0	0	0	0	0
M	124	74	99	69	117	51	77	51	103	70	0	0	0	1	0	0	0	0	0	0
M	120	70	123	67	114	64	110	67	113	71	0	0	0	0	0	0	0	0	0	0
M	105	74	111	71	137	85	91	56	85	50	0	0	0	0	1	0	0	0	0	0
M	110	60	82	42	117	63	123	77	124	78	0	1	0	0	0	0	1	0	0	0
M	135	91	89	52	117	62	114	44	104	49	0	1	0	0	0	0	0	0	1	1
M	157	103	136	85	135	109	138	91	127	80	0	0	0	0	0	0	0	0	0	0
M	132	69	103	60	114	63	95	51	114	57	0	0	0	0	0	0	0	0	0	0
M	135	99	125	72	126	83	91	55	107	65	0	0	0	0	0	0	0	0	0	0
M	113	74	119	72	103	73	110	70	120	57	0	0	0	0	0	0	0	0	0	0
M	102	71	97	57	112	59	106	56	115	72	0	0	0	0	0	0	0	0	0	0
M	115	67	99	56	122	72	104	58	120	76	0	0	0	0	0	0	0	0	0	0
M	123	76	111	67	112	74	95	57	115	65	0	0	0	0	0	0	0	0	0	0
M	113	70	117	79	84	60	103	41	95	36	0	0	1	0	0	0	0	0	1	1
M	145	93	125	89	121	78	113	62	122	47	0	0	0	0	0	0	0	0	0	1
M	116	71	69	48	108	60	95	51	102	58	0	1	0	0	0	0	1	0	0	0
M	123	52	144	84	150	75	130	68	142	73	0	0	0	0	0	0	0	0	0	0
M	109	77	115	74	112	52	93	53	93	53	0	0	0	0	0	0	0	0	0	0
M	122	68	107	60	125	72	110	64	106	65	0	0	0	0	0	0	0	0	0	0
M	121	67	117	39	111	45	83	63	97	72	0	0	0	1	0	0	1	1	0	0
M	120	80	107	62	100	50	105	60	120	70	0	0	0	0	0	0	0	0	0	0
M	127	88	110	68	112	75	110	64	102	60	0	0	0	0	0	0	0	0	0	0

M	155	91	77	47	115	67	113	65	126	74	0	1	0	0	0	0	1	0	0	0
M	121	88	78	53	111	62	104	52	102	65	0	78	0	0	0	0	1	0	0	0
P	105	59	96	58	110	87	82	45	107	63	0	0	0	1	0	0	0	0	1	0
P	130	87	71	44	114	72	101	50	108	64	0	1	0	0	0	0	1	0	0	0
P	168	98	151	101	135	105	153	86	146	87	0	0	0	0	0	0	0	0	0	0
P	137	85	131	79	124	97	95	52	102	63	0	0	0	0	0	0	0	0	0	0
P	130	77	136	74	130	78	107	54	120	51	0	0	0	0	0	0	0	0	0	0
P	143	94	136	93	173	142	146	90	152	97	0	0	0	0	0	0	0	0	0	0
P	120	70	129	76	127	70	119	64	131	82	0	0	0	0	0	0	0	0	0	0
P	160	80	120	60	144	70	140	70	130	80	0	0	0	0	0	0	0	0	0	0
P	135	86	101	67	142	85	103	56	112	74	0	0	0	0	0	0	0	0	0	0
P	170	60	91	44	119	63	82	45	88	43	0	0	0	1	1	0	1	0	1	1
P	157	82	161	75	158	81	159	98	172	96	0	0	0	0	0	0	0	0	0	0
P	114	74	84	49	116	72	88	48	110	70	0	1	0	1	0	0	0	0	0	0
P	139	91	112	65	120	70	92	49	106	62	0	0	0	0	0	0	0	0	1	0
P	143	59	127	80	172	80	174	90	176	185	0	0	0	0	0	0	0	0	0	0
P	129	75	114	62	128	69	127	63	125	63	0	0	0	0	0	0	0	0	0	0
P	120	77	136	86	131	87	106	65	111	71	0	0	0	0	0	0	0	0	0	0
P	128	80	96	50	112	71	121	65	124	73	0	0	0	0	0	0	0	0	0	0
P	155	93	134	88	152	90	106	64	114	79	0	0	0	0	0	0	0	0	0	0
P	118	86	112	67	118	69	107	60	110	73	0	0	0	0	0	0	0	0	0	0
P	143	65	134	65	143	60	120	62	116	57	0	0	0	0	0	0	0	0	0	0
P	131	84	131	82	129	82	118	62	119	69	0	0	0	0	0	0	0	0	0	0
P	136	98	154	86	146	77	102	51	111	68	0	0	0	0	0	0	0	0	0	0
P	130	87	121	83	117	70	82	52	109	74	0	0	0	1	0	0	0	0	0	0
P	115	80	107	69	130	80	130	82	95	53	0	0	0	0	0	0	0	0	0	0
P	112	78	130	64	113	76	96	53	104	62	0	0	0	0	0	0	0	0	0	0
P	143	92	76	52	121	78	110	60	108	60	0	1	0	0	0	0	0	0	0	0
P	104	59	98	60	112	88	85	47	108	60	0	0	0	1	0	0	0	0	1	0
PLACEBO	122	70	123	75	109	74	104	56	136	78	0	0	0	0	0	0	0	0	0	0
PLACEBO	126	80	116	63	126	69	90	44	112	70	0	0	0	0	0	0	0	0	1	0

PLACEBO	122	77	75	39	124	49	105	47	110	67	0	1	0	0	0	0	1	0	1	0
PLACEBO	125	68	130	74	124	69	92	54	104	62	0	0	0	0	0	0	0	0	0	0
PLACEBO	113	84	89	57	86	62	90	65	126	79	0	1	1	0	0	0	0	0	0	0
PLACEBO	133	84	92	50	134	83	87	52	107	56	0	0	0	1	0	0	0	0	0	0
PLACEBO	115	74	100	38	109	59	106	52	107	49	0	0	0	0	0	0	1	0	0	1
PLACEBO	135	85	95	50	127	80	120	50	130	65	0	0	0	0	0	0	0	0	0	0
PLACEBO	121	78	109	63	138	83	152	62	114	52	0	0	0	0	0	0	0	0	0	0
PLACEBO	116	89	116	66	128	57	117	84	109	68	0	0	0	0	0	0	0	0	0	0
PLACEBO	110	73	93	70	109	69	103	64	107	68	0	0	0	0	0	0	0	0	0	0
PLACEBO	110	70	90	50	108	51	100	60	110	70	0	0	0	0	0	0	0	0	0	0
PLACEBO	118	74	100	54	100	62	98	49	98	54	0	0	0	0	0	0	0	0	1	0
PLACEBO	135	86	106	72	98	57	89	48	107	64	0	0	0	1	0	0	0	0	1	0
PLACEBO	129	83	92	55	114	66	117	64	122	65	0	0	0	0	0	0	0	0	0	0
PLACEBO	135	91	110	53	130	84	147	83	137	91	0	0	0	0	0	0	0	0	0	0
PLACEBO	138	74	100	43	102	58	117	58	123	67	0	0	0	0	0	0	1	0	0	0
PLACEBO	126	69	130	75	120	68	93	55	105	64	0	0	0	0	0	0	0	0	0	0
PLACEBO	134	84	82	40	126	79	120	60	128	66	0	1	0	0	0	0	1	0	0	0
PLACEBO	112	75	100	60	125	50	106	48	110	68	0	0	0	0	0	0	0	0	1	0
PLACEBO	125	80	115	65	125	70	90	45	110	65	0	0	0	0	0	0	0	0	1	0
PLACEBO	128	80	80	50	115	65	117	65	120	65	0	1	0	0	0	0	0	0	0	0
PLACEBO	115	75	100	60	108	58	107	52	106	50	0	0	0	0	0	0	0	0	0	0
PLACEBO	120	70	124	76	109	75	105	56	135	78	0	0	0	0	0	0	0	0	0	0
PLACEBO	115	90	115	65	128	55	118	85	110	68	0	0	0	0	0	0	0	0	0	0
PLACEBO	108	66	134	67	127	76	89	49	102	60	0	0	0	1	0	0	0	0	1	0
PLACEBO	139	76	79	37	91	53	114	47	114	47	0	1	0	0	0	0	0	0	1	1
PROMET	131	84	122	73	139	74	128	65	113	69	0	0	0	0	0	0	0	0	0	0
PROMET	136	85	96	59	95	60	105	62	126	79	0	0	0	0	0	0	0	0	0	0
PROMET	129	80	137	79	73	24	106	62	115	69	0	0	1	0	0	0	0	1	0	0
PROMET	125	79	110	68	114	77	100	56	96	64	0	0	0	0	0	0	0	0	0	0
PROMET	131	87	80	57	89	48	104	47	107	64	0	1	1	0	0	0	0	1	1	0
PROMET	128	89	112	70	117	78	117	73	128	55	0	0	0	0	0	0	0	0	0	0

PROMET	120	74	135	75	111	69	92	52	109	57	0	0	0	0	0	0	0	0	0	0
PROMET	130	78	129	63	122	55	96	46	94	49	0	0	0	0	0	0	0	0	1	1
PROMET	132	78	107	49	120	60	102	59	117	71	0	0	0	0	0	0	1	0	0	0
PROMET	124	75	100	60	111	63	85	50	101	63	0	0	0	1	0	0	0	0	0	0
PROMET	146	92	138	79	122	81	126	70	106	71	0	0	0	0	0	0	0	0	0	0
PROMET	135	90	127	80	120	68	117	64	108	57	0	0	0	0	0	0	0	0	0	0
PROMET	144	77	141	88	121	67	82	48	96	62	0	0	0	1	0	0	0	0	1	0
PROMET	118	69	103	66	101	57	96	51	104	63	0	0	0	0	0	0	0	0	0	0
PROMET	113	62	127	78	112	73	111	69	105	63	0	0	0	0	0	0	0	0	0	0
PROMET	120	70	105	66	90	56	90	47	110	73	0	0	0	0	0	0	0	0	1	0
PROMET	146	79	90	44	130	68	124	59	122	66	0	1	0	0	0	0	0	0	0	0
PROMET	131	45	151	79	125	80	109	60	113	59	0	0	0	0	0	1	0	0	0	0
PROMET	123	88	80	45	117	60	96	36	130	78	0	1	0	0	0	0	1	0	1	0
PROMET	132	85	103	62	88	46	87	41	89	51	0	0	1	1	1	0	0	1	1	0
PROMET	128	77	128	62	120	56	98	46	96	50	0	0	0	0	0	0	0	0	1	0
PROMET	122	75	136	74	113	70	93	50	110	58	0	0	0	0	0	0	0	0	0	0
PROMET	133	88	105	58	91	50	105	48	108	65	0	0	0	0	0	0	0	0	1	0
PROMET	124	78	111	69	113	76	101	57	98	65	0	0	0	0	0	0	0	0	0	0
PROMET	132	85	122	75	138	75	127	64	114	69	0	0	0	0	0	0	0	0	0	0
PROMET	120	60	124	80	122	72	104	53	125	68	0	0	0	0	0	0	0	0	0	0
PROMET	140	80	121	78	108	70	102	60	126	66	0	0	0	0	0	0	0	0	0	0
PROMET	110	80	114	70	114	70	96	53	110	70	0	0	0	0	0	0	0	0	0	0
